# Early functional improvements using continuous passive motion therapy after angular-stable plate osteosynthesis of proximal humerus fractures – results of a prospective, randomized trial

**DOI:** 10.1186/s13018-024-04804-x

**Published:** 2024-05-28

**Authors:** Eric Tille, Franz Lorenz, Franziska Beyer, Antonia Schlüßler, Achim Biewener, Jörg Nowotny

**Affiliations:** University Center of Orthopaedic, Trauma and Plastic Surgery, University Hospital Carl Gustav Carus, TU Dresden, Fetscherst. 74, 01307 Dresden, Germany

**Keywords:** Shoulder, Proximal humerus fracture, Continuous passive motion, CPM, Plate osteosynthesis, Rehabilitation, Patient-reported outcome

## Abstract

**Background:**

The use of continuous passive motion therapy (CPM) has led to promising results in the early phase of rehabilitation after surgical treatment of rotator cuff tears and arthrolysis of the elbow. However, its use has not been proven in other pathologies of the upper extremity. Therefore, the aim of the underlying study was to evaluate the use of CPM therapy after plate osteosynthesis of proximal humeral fractures.

**Methods:**

95 patients with isolated proximal humerus fractures were enrolled in a prospective, randomized study. Patients were assigned to a treatment group with (*n* = 48, CPM) or without CPM therapy (*n* = 47, CG). Four patients (2 of each cohort) violated the study protocol and were excluded. CPM therapy was used for 6 weeks after surgery 2–3 times daily. Functional (range of motion) and patient reported outcomes (PROM, Constant Score [CSS], QuickDASH, subjective shoulder value [SSV], pain on visual analogue scale [VAS]) were evaluated at 6 weeks, 3 and 12months. 60 patients completed the 1-year follow-up.

**Results:**

The average patient age was 65.3 years (min: 27, max: 88, SD: ± 14.7). Seventy-two patients were female (79%). There was no difference regarding injury severity (2/3/4 part-fracture: 6/32/7 vs. 9/26/11, *p* = 0.867) and sex (*p* = 0.08). However, patients in the CPM group were significantly younger (CPM: 67 [min: 34, max: 82], CG: 74 [min: 27, max: 88], *p* = 0.032). After 6 weeks we observed a better range of motion for forward flexion (CPM: 90° [min: 50°, max: 180°] vs. CG: 80° [min: 20°, max: 170°] *p* = 0.035) and abduction (CPM: 80° [min: 40°, max: 180°] vs. CG: 70° [min: 20°, max: 180°], *p* = 0.048) in the CPM group. There was no difference regarding the further planes of motion or the assessed PROMs at 6 weeks. At 3 and 12 months the results between the treatment groups equalized with no further significant differences.

**Conclusion:**

The treatment with CPM increases the range of motion after plate osteosynthesis of proximal humerus fractures in the first 6 weeks after surgery. This effect is not sustained after 3 and 12months. The evaluated PROMs are not being influenced by CPM therapy. Hence the results of this prospective randomized study suggest that CPM can be a beneficial asset in the early period of rehabilitation after proximal humerus plate osteosynthesis.

**Trial registration:**

The study protocol was registered in the US National Institutes of Health’s database (http://www.clinicaltrials.gov) registry under NCT 05952622.

## Background

The benefits of continuous passive motion therapy (CPM) have been proven by multiple studies in the early rehabilitation phase after surgical treatment of rotator cuff tears and arthrolysis of the elbow [1–4]. This seems to be easily comprehensible since the earlier mobilization is preventing scarring of the tendons and the joint [5]. While CPM therapy has also been used in the mobilization of the knee (i.e., after reconstruction of the anterior cruciate ligament or following total knee arthroplasty) there have been no reports for its use in other pathologies of the upper extremity [6, 7].

Proximal humerus fractures (PHF) account for up to 10% of all fractures [8–10]. In Germany approximately 60,000 PHF have been reported in 2019 [9]. The associated health care related and economic burden due to hospitalization, medical treatment, aftercare and temporary loss of work force is immense [11, 12]. While younger patients (< 65 years) typically tend to have a better recovery, their injury-associated absence is still 47 days on average [12].

While PHF – depending on the associated trauma - can occur at any age, a higher prevalence has been observed in the elderly [11]. Because of the expected demographic change - leading to a larger geriatric population with a higher activity level and a growing functional demand - prior studies have demonstrated an additional increase in incidence of fragility fractures, including the proximal humerus. In the elderly, it is estimated that up to 20% of all osteoporotic fractures are PHF [9]. In this highly vulnerable group, PHF can have an additional social impact leading to loss of independence, inpatient hospitalization and the need of a nursing home [11]. Meanwhile younger patients need a fast convalescence after PHF in order to be able to return to work as quickly as possible and to reduce periods of absence to a minimum.

In the past decades the surgical techniques addressing these injuries have been evolving. A trend towards conservative or endoprosthetic replacement has been noticed, especially in geriatric patients [13]. However, only few studies have focused upon the rehabilitation process [14–16]. Yet the postoperative aftercare is an essential component for the success of the surgical treatment.

Under consideration of the aforementioned facts, the question was raised whether treatment with CPM could be beneficial after angle-stable plate osteosynthesis of the proximal humerus. We hypothesized that a rehabilitation protocol, including the use of CPM, is beneficial towards the functional and patient-reported outcomes.

## Materials and methods

The study protocol was registered in the US National Institute of Health`s database registry (http://www.clinicaltrials.gov) under NCT 05952622. After institutional review board approval (EK 443112018) a prospective, randomized-controlled trial was initiated. After informed consent was obtained, a total of 95 patients suffering from an acute fracture of the proximal humerus treated with open reduction and plate osteosynthesis were enrolled between April 1st, 2018 and February 28th, 2022. Treatment decision was based upon patient-individual criteria including but not limited to age, comorbidities, expected compliance, functional demand as well as injury-specific factors such as dislocation, severity and bone quality. Exclusion criteria comprised patients treated with arthroplasty, additional ipsilateral fracture of the upper extremity (i.e. distal radius fracture), traumatic brain injury, brachial plexus lesion with and without nerve palsy, addictive diseases (i.e. alcohol abuse) and reduced compliance. Patients received plate osteosynthesis using a proximal humerus interlocking system (PHILOS, Fa. DePuy Synthes) and tension banding of the tubercula / rotator cuff if necessary. In cases of reduced bone quality, additional cement augmentation was performed. Surgery was carried out under general anesthesia with or without a temporary regional nerve blockade as pain treatment. Aftercare followed a standardized rehabilitation protocol. Following surgery patients were initially immobilized with an orthesis (Gilchrist) for either 2 weeks (2-part fractures) or 3 weeks (3- and 4-part fractures, reduced bone quality). Hereafter patients were allowed to move the arm actively without limitations. Weight-bearing was restricted to 0.5 kg for a total of 6 weeks. Starting on day 7 all patients underwent professional physical therapy, initially consisting of pendulum exercises and passive movement. In the course of time therapy was escalated to active movement and strengthening exercise. All patients received at least 18 sessions of physical therapy (usually 2–3 times per week, 30–40 min).

Additionally, patients were assigned randomly to one of two groups, either receiving treatment with a continuous passive motion device (KINETEC® Centura, Fa. MTR Medizintechnik Rostock, Germany, CPM group) or not (CG group). Randomization was done consecutively according to a software generated randomization list depending on time of initial clinical presentation. 48 patients were assigned to the CPM group and 47 patients to the CG group. The CPM treatment protocol included training of abduction and forward flexion starting immediately after the recommended immobilization period for 6 weeks with 2–3 sessions per day. The range of motion was increased gradually each week depending on the patient’s individual progress and pain.

**Fig. 1:**
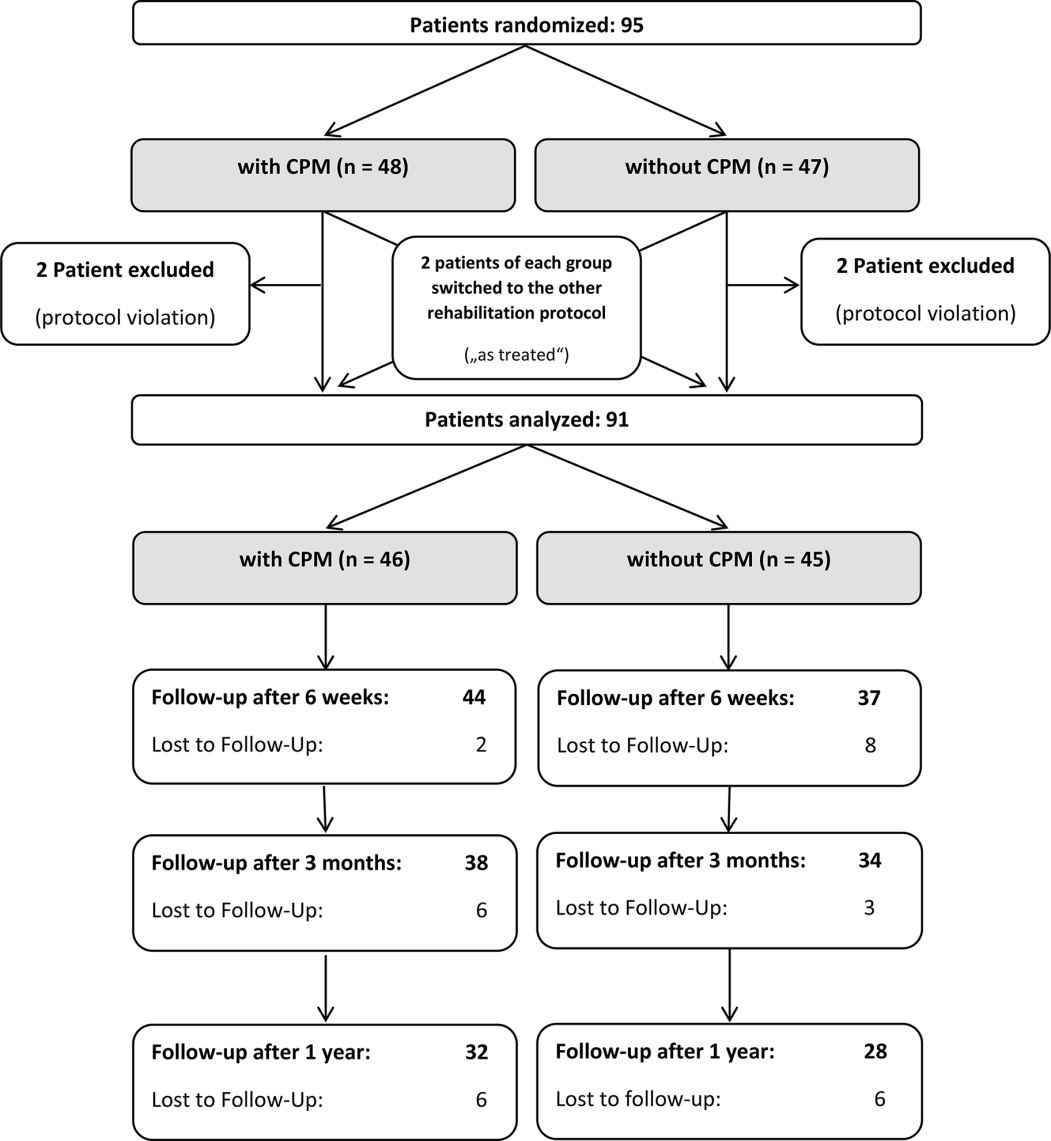
Flowchart of study patients enrollment and Follow-Up.

Four patients, 2 treated with and 2 patients treated without CPM, violated the study protocol and were excluded. Therefore, the data of 91 patients was analyzed (Fig. 1).

Prior to surgery, demographic factors including age, sex, dominant hand, body weight, body mass index (BMI) and American Society of Anesthesiologists Score (ASA) were assessed. Furthermore, the fracture morphology and side of the involved shoulder was analyzed. Patients were evaluated by a study nurse or a physician who was not directly involved in the surgical treatment clinically at 6 weeks, 3 months and 1 year after surgery. The assessors were not blinded. Functional (range of motion [ROM]) and patient-reported outcome measures (PROM) including Constant Shoulder Score (CSS) and Quick Disabilities of Shoulder, Arm and Hand Score (qDASH) as well as the subjective shoulder value (SSV) and pain both rated on a visual analogue scale (VAS) were assessed. Due to a defective spring balance, no reliable comparability could be ensured regarding the strength measurements of the CSS subcategory. We therefore only compared the subcategories of pain, activities of daily living, and movement. Sixty patients completed the 1-year follow up (FU) (Fig. 1).

### Statistical analysis

Sample size calculation was based on abduction of the shoulder. To detect a difference of 7° (estimated standard deviation [SD] of 10°) with a power of 0.8 and a significance level of *p* < 0.05, a minimum of 33 patients per group were necessary.

Data description was based on means and SD for continuous values and absolute and relative frequencies for categorical values. Comparisons between treatment groups were done ‘as treated’ by Mann-Whitney-U-Test for continuous values and chi-square test for categorical values. Significance level was set at *p* < 0.05. The software SPSS (release 26 for Windows) was used for data analysis.

## Results

The average patient age was 65.3 years (min: 27, max: 88, SD: ±14.7). Seventy-two patients were female (79%). In 44 cases (48.4%) the right shoulder was injured. There was no significant difference regarding injury severity (2/3/4 part-fracture: CPM 9/26/11 vs. CG 6/32/7, *p* = 0.867) and sex (CPM m/w 13/33 vs. CG m/w 6/39, *p* = 0.08). However, patients in the CPM group were significantly younger (CPM: 67.0 [min: 34, max: 82, SD: 13.1], CG: 74 [min: 27, max: 88, SD: 15.2], *p* = 0.03). The further demographic parameters revealed no difference between treatment groups (Table 1).

**Table 1:** Demographic factors. Values given as median with range. *p* < 0.05

	Without CPM (*n* = 45)	With CPM (*n* = 46)	*p*-value
Age at surgery [years]	74.0 (27; 88)	67.0 (34; 82)	**0.032**
Female gender	86.7% (*n* = 39)	71.7% (*n* = 33)	0.08
Weight [kg]	79.0 (56; 130)	76.0 (60; 120)	0.271
BMI [kg/m²]	28.1 (20.1; 52.1)	27.3 (18.1; 45.7)	0.611
Involved shoulder			
Left	25 (55.6%)	22 (47.8%)	0.601
Right	20 (44.4%)	24 (52.2%)	
Dominant hand			
Left	1 (2.2%)	3 (6.5%)	
Right	44 (97.8%)	41 (89.1%)	0.321
No preference	0 (0.0%)	1 (2.2%)	
Unknown	0 (0.0%)	1 (2.2%)	
Fracture morphology			
2part-fracture	6 (13.3%)	9 (19.6%)	
3part-fracture	32 (71.1%)	26 (56.5%)	0.867
4part-fracture	7 (15.6%)	11 (23.9%)	
With additional head-split	1 (2.2%)	1 (2.2%)	
ASA Score			
ASA grade 1 or 2	27 (60.0%)	37 (80.0%)	
ASA grade 3 or 4	18 (40.0%)	9 (20.0%)	0.097

At the 1-year FU, one patient died due to other medical conditions. Furthermore, 13 were lost to follow-up. Seven patients presented with complications. In 4 patients treated without CPM we observed failure of the osteosynthesis (CPM 0 vs. CG 4, *p* = 0.039). Three patients treated with CPM suffered from necrosis of the humeral head (CPM 3 vs. CG 0, *p* = 0.08). In addition, 13 patients (CPM 4; CG 9; *p* = 0.123) reported insufficient functional recovery in terms of ROM not reaching 90° of forward flexion and abduction 3–6 months after initial surgery and therefore needed plate removal and/or arthrolysis. Overall, 32 patients in the group with CPM treatment and 28 patients in the group without CPM treatment completed the 1-year FU.

**Fig. 2:**
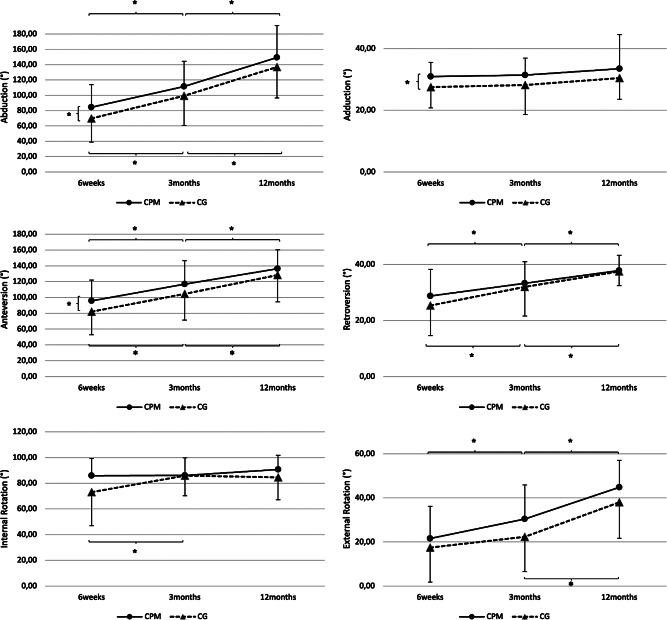
Longitudinal evaluation of the functional results. Comparison of range of motion at 12-month follow-up between patients treated with standard rehabilitation protocol (CG) and rehabilitation including continuous passive motion therapy (CPM). Significant differences between the groups were found at the 6 weeks FU for abduction, adduction and forward flexion. Significant differences between timepoints are marked with * (*p* < 0.05)

After 6 weeks we observed a significantly better range of motion for forward flexion (CPM: 90° [min: 50°, max: 180°] vs. CG: 80° [min: 20°, max: 170°], *p* = 0.035), adduction (CPM: 30° [min: 20°, max: 50°] vs. CG: 30° [min: 10°, max: 40°], *p* = 0.049) and abduction (CPM: 80° [min: 40°, max: 180°] vs. CG: 70° [min: 20°, max: 180°], *p* = 0.048) in the CPM group. There was no difference regarding the further planes of motion. At the 3- and 12-month FU the results between treatment groups equalized with no further significant differences (Table 2). Figure 2 displays the functional results until the 12-month FU.

**Table 2:** Functional results at the various FU timepoints. Values given as mean with range. *p* < 0.05

	without CPM (*n* = 28)	with CPM (*n* = 32)	*p*-value
Abduction			
6 weeks	70 (20; 170)	80 (40; 180)	**0.048**
3 months	90 (30; 180)	100 (70; 180)	0.167
12 months	140 (40; 180)	180 (70;180)	0.131
Adduction			
6 weeks	30 (10; 40)	30 (20; 50)	**0.049**
3 months	30 (5; 60)	30 (20; 50)	0.056
12 months	30 (3; 40)	30 (30; 90)	0.640
Forward flexion			
6 weeks	80 (20; 170)	90 (50; 180)	**0.035**
3 months	103 (30; 170)	120 (10: 160)	0.105
12 months	140 (30; 170)	150 (70; 180)	0.519
Backward extension			
6 weeks	23 (0; 40)	30 (10; 50)	0.289
3 months	38 (0;40)	30 (20; 50)	0.994
12 months	40 (0; 40)	40 (20; 50)	0.849
External rotation			
6 weeks	10 (-10; 60)	20 (0; 50)	0.246
3 months	23 (0; 50)	30 (0; 70)	0.056
12 months	48 (5; 60)	50 (10; 70)	0.132
Internal Rotation			
6 weeks	90 (10; 95)	90 (40; 95)	0.078
3 months	90 (40; 95)	90 (20; 95)	0.803
12 months	93 (40; 95)	95 (40; 95)	0.085

**Fig. 3:**
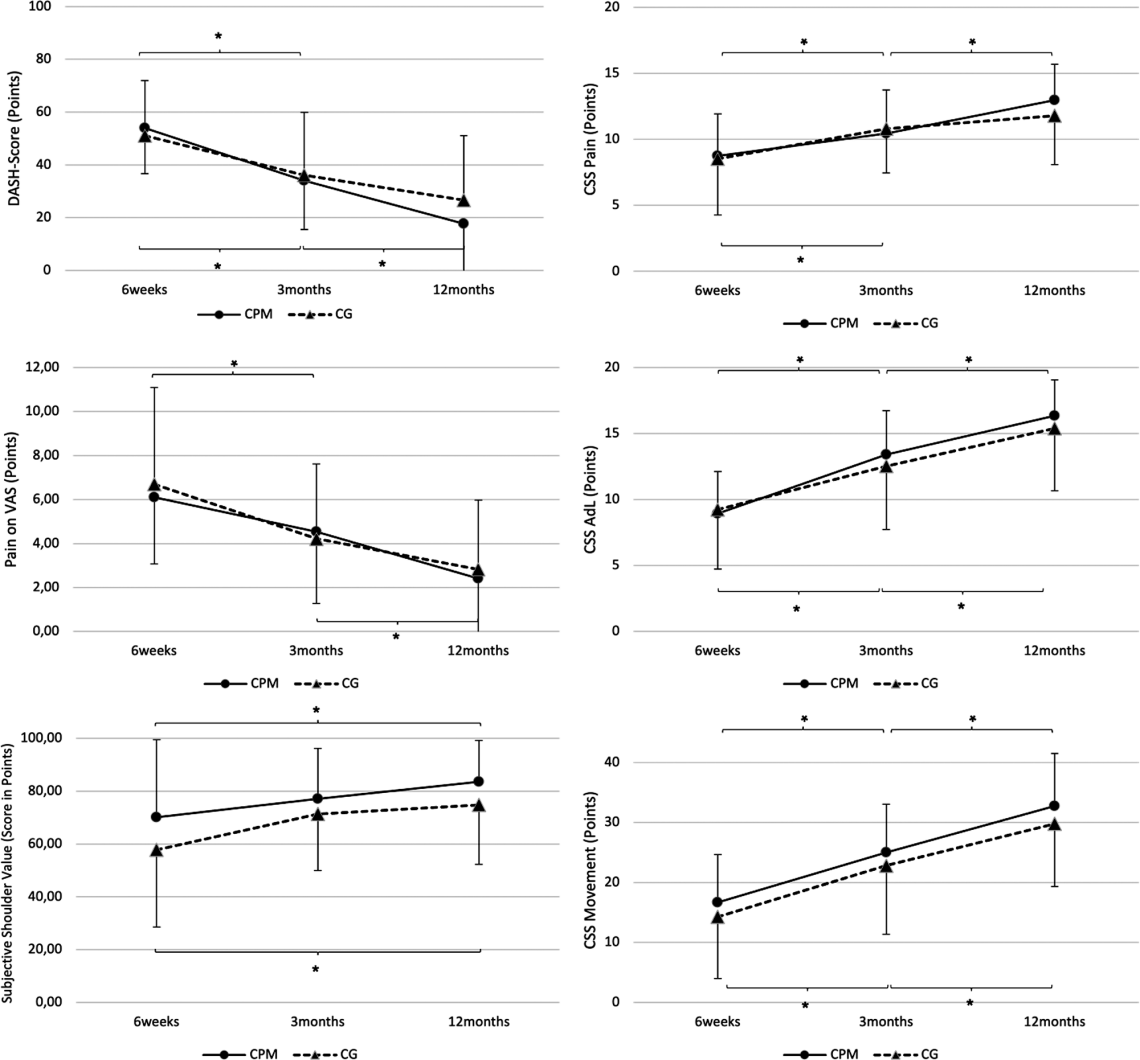
Longitudinal comparison of patient-reported outcome. Parameters evaluated are [A] qDASH-Score, [B] Pain on Visual Analogue Scale (VAS), [C] Subjective Shoulder Value (SSV), [D-F] Subcategories of Constant Shoulder Score (CSS), specifically [D] Pain, [E] Activities of Daily Living (AdL), [F] Movement. A significant improvement between timepoints can be seen for almost every parameter (*, *p* < 0.05). Between groups there were no significant differences at any timepoint

Regarding the patient-reported outcome we found no statistically significant differences at any given FU for the evaluated parameters (Tables 3 and 4). For the movement subcategory of the CSS a trend towards a better result of the CPM group was noticed (Table 4). This trend however was not significant (*p* = 0.081). The time course of the PROMs is displayed in Fig. 3.

**Table 3:** Patient-reported outcome at the various FU timepoints. Values given as mean with range. *p* < 0.05

	without CPM (*n* = 28)	with CPM (*n* = 32)	*p*-value
Subjective Shoulder Value (SSV) [0-100]			
6 weeks	50.0 (10; 100)	75.0 (20; 100)	0.182
3 months	72.5 (20; 100)	80.0 (45; 100)	0.331
12 months	80.0 (15; 100)	90.0 (40; 100)	0.217
Pain on VAS [0–15]			
6 weeks	6.5 (0; 15)	7.0 (0; 11)	0.923
3 months	4.0 (0; 14)	4.0 (0; 13)	0.648
12 months	1.0 (0; 9)	1 (0; 14)	0.566
DASH-Score [0-100]			
6 weeks	50.0 (13.6; 88.6)	54.6 (20.5; 88.6)	0.553
3 months	30.7 (0.0; 81.8)	31.8 (2.3; 72.7)	0.970
12 months	22.7 (0.0; 75.0)	9.1 (0.0; 77.3)	0.229

**Table 4:** Outcome of the subcategories of the CSS at the various FU timepoints. Values given as mean with range. *p* < 0.05

	without CPM (*n* = 28)	with CPM (*n* = 32)	*p*-value
CSS: Pain [0–15, max: 15]			
6 weeks	8.5 [0.0, 15.0]	8.0 [4.0; 15.0]	0.923
3 months	11.0 [1.0; 15.0]	11.0 [2.0; 15.0]	0.648
12 months	14.0 [6.0; 15.0]	14.0 [1.0; 15.0]	0.566
CSS: Activities of Daily Living [0–20, max: 20]			
6 weeks	10.0 [2.0; 20.0]	9.0 [2.0; 15.0]	0.815
3 months	13.0 [3.0; 20.0]	13.5 [2.0; 20.0]	0.392
12 months	16.0 [6.0; 20.0]	18.0 [4.0; 20.0]	0.319
CSS: Movement [0–40, max: 40]			
6 weeks	10.0 [0.0; 40.0]	16.0 [8.0; 30.0]	0.081
3 months	22.0 [4.0; 40.0]	27.0 [10.0; 40.0]	0.154
12 months	32.0 [8.0; 40.0]	38.0 [8.0; 40.0]	0.263

Furthermore, univariate linear regression displayed no significant result for the evaluated demographic factors.

## Discussion

The case number for PHF is rising substantially due to demographic changes and the higher level of activity in the elderly. An epidemiological study of Kim et al. stated a prevalence of 183,400 PHF in the United States for the year 2008 [17]. In Germany, with a population of only a fourth of the US, 61,606 PHF have been registered in the year 2019. Despite the already high case numbers, there has been an increase in prevalence between 2009 and 2019 by about 10% [9].

Most fractures of the proximal humerus are fragility fractures of the elderly. It is estimated that nearly 20% of all fragility fractures are injuries of the proximal humerus [9]. Former studies by Dietrich et al. have shown a continuous increase of incidence in association to the life decade, with the highest risk at an age older than 70 years [8, 9]. Further risk factors comprise female gender and reduced bone density or diagnosed osteoporosis [18, 19]. This is similar to our results which display an average patient age of 65.1 ± 14.8 years and an almost four times higher prevalence in females (male : female ratio = 1 : 3.8). Due to the high case count, PHF cause a tremendous burden to health care systems. A Canadian study estimated total treatment costs for fragility fractures to be about $2.3 billion in 2010 [20]. Treatment costs include hospitalization, surgical and/or non-surgical therapy, ambulant aftercare, physiotherapy as well as ambulant and inpatient nursing services. In a study by Maravic et al. it was estimated that hospital treatment of PHF caused €36.6 million in health care costs in France in 2009 [19]. Yet even with sufficient treatment there is a significantly higher rehospitalization and mortality rate after PHF, especially in the first year after treatment as Curtin et al. and Maravic et al. have shown [19, 21].

Alongside the fact that most PHF occur at a higher age, Dietrich et al. have also observed an increase in case numbers for younger patients of all age groups [9]. This is especially relevant since in addition to health care costs in patients prior to retirement age a temporary or permanent loss of workforce and earnings must be considered. This leads to an auxiliary economic burden. Further costs arise due to a prolonged return-to-work after PHF, even after surgical treatment. Studies estimate that, depending on the occupation the average downtime is 42 days for professions with a low-physical demand (i.e., office-workers) and 118 days for professions with a high physical demand [12]. Inauen et al. demonstrated that normal scores regarding quality-of-life following PHF were not achieved until 6 months after trauma. However, depending on the fracture type the recovery can be delayed, since more complex injuries present with a slower betterment [22]. The best possible treatment and aftercare therefore seems to be a necessity.

Throughout the past years and decades many studies have tried to elucidate the question whether conservative/non-operative, reconstructive (plate or nail osteosynthesis) or replacement (arthroplasty) surgery should be the gold standard in the treatment of PHF [23–26]. The potential benefits of surgical treatment include the missing need for pronounced immobilization of the limb, generating stability, and making early rehabilitation possible, therefore preventing scarring, joint stiffness and (ideally) shortening recovery time [27]. Yet none of the above-mentioned treatment options have proven a clear medical or economic advantage [28–30]. Treatment choice therefore remains a highly individual decision.

While treatment itself has been investigated in depth, only few studies have focused upon rehabilitation and aftercare [31]. In 2021, a study of Rohun et al. concluded that there is “only limited publicly available information on the rehabilitation following PHF“[32]. This is unfortunate since rehabilitation is an essential part of the surgical treatment. In 2007, Hodgeson et al. suggested immediate physical therapy to prevent the harmful effects of prolonged immobilization [15, 16]. This is consistent with previous results from Kristiansen et al., which found a shorter period of immobilization (1 week) to be beneficial towards early recovery of range of motion [14]. In a recent approach, Aguado et al. evaluated a home-based rehabilitation protocol, which lead to promising results and a high level of satisfaction [33]. Considering the availability of physiotherapists especially in rural areas, the current lack of skilled labour in many western countries and the limited mobility of older patients, this might be a sustainable approach.

New rehabilitation methods involve robotic or telerehabilitation programs. The evidence however is currently low. Cabana et al. initiated a study comparing telerehabilitation to a face-to-face training program in 2016 [34]. The results of this trial however have not yet been published. Schwickert et al. presented data of a robotic-assisted rehabilitation for geriatric patients. Despite a low number of participants, the study displayed a high acceptance and an increase in functional results [35]. A similar approach by Nerz et al. is currently being evaluated in a randomized controlled study initiated in 2017 by the same research group comparing robot-assisted training to conventional rehabilitation [36]. Finley et al. described an occupational-based rehabilitation model in a case report of a 4-part fracture with good results [37]. Yet there have been no studies evaluating the effect of CPM therapy in the aftercare of PHF. This is albeit the fact that the use of CPM therapy has been proven beneficial for aftercare in knee surgery and some entities of shoulder surgery (rotator cuff tears, stiffness) [38]. In the 1990’s, Salter et al. have proven with their experimental studies that CPM therapy enhances the metabolism of the joint, improves the resorption of effusions and may prevent joint stiffness and secondary arthrosis [39–41]. In line with these results, our study shows a significant beneficial effect of CPM therapy with an improved abduction, adduction and forward flexion within the first six weeks after surgical treatment. In the further FU, the functional results between study groups did not differ significantly anymore. A possible reason might be that CPM therapy was terminated after 6 weeks. Also, CPM therapy does not increase the overall ROM that can be achieved, but rather facilitates a faster rehabilitation in the early phase after surgical treatment. Garofalo et al. who examined the use of CPM therapy after rotator cuff repair in 2010 described similar results with an initially faster rehabilitation but no persisting differences at the one-year FU [4]. This is a critical information especially for vulnerable patient groups or patients with a high functional demand in the early phase after PHF. In contrast to these positive functional results, we observed no significant differences related to the evaluated patient-reported outcome parameters. While the result for the subcategories of the CSS were not significant, we observed a trend towards a better outcome for the movement category with *p* = 0.081. A potential cause of why the PROMs do not display any significant results might be that the differences in the scores used are too small to be clinically noticeable. In 2013, Kukkonen et al. described a threshold for a minimal clinically important difference (MCID) for the CSS with 10.4 points [42]. Dabija et al. found the MCID for CSS after PHF to be 5.4–11.6 and for the DASH score to be 8.1–13.0 [43]. Generally, the subjective perception measured with the PROMs is closely linked to the functional results. Yet even though there is a significant improvement of the ROM, the beneficial effect might be too small to make a difference in the patient’s daily life. Taking under account the patients age between 60 and 70 years, Simovitch et al. described a MCID of 17.2° ± 6.8° for forward flexion and 7.2° ± 5.9° for abduction [44]. The described changes might therefore merely not be perceivable by the patients. Another reason could be that the number of patients enrolled in this study is too small to detect differences between the study groups.

Despite the insignificant patient reported results the underlying study proves for the first time that CPM therapy can facilitate a faster functional rehabilitation after osteosynthetic PHF treatment in the early period of rehabilitation.

### Limitations

Limitations of this study include the small sample size and the monocentric design. Also, the aforementioned difference in patient age might attribute to a bias in functional and patient-reported outcome. Usually, younger patients have a higher potential to regain better functionality after fracture treatment. Since the further demographic parameters (gender, comorbidity, BMI, fracture morphology, etc.) did not reveal any statistic differences we would still consider the different cohorts to be comparable. Another limitation applies to the physical rehabilitation program. While all patients received at least 18 sessions of physiotherapy these have not been standardized due to the different severity of the injury. Patients may therefore have received a heterogeneous physical therapy concerning quality and intensity. Also, the compliance regarding the use of the CPM, meaning whether CPM was used as advised, could not be assessed objectively by the investigators. Especially older patients struggle to comply with medical recommendations such as immobilization and physiotherapy. Fleischhacker et al. showed in a recent study that only 30–50% terminated orthosis and received physiotherapy as planned [45]. Potentially technical solutions tracking the shoulder activity as evaluated by Hartleer et al. could help with this issue in the future [46].

## Conclusion

Postoperative treatment with a CPM device following angle-stable plate osteosynthesis of PHF results in a slightly better functional range of motion 6 weeks after surgery. Its use could therefore be an asset towards a faster rehabilitation especially in vulnerable patient groups with early return to work or a high functional demand. Yet the beneficial results are not sustained over time and do not seem to translate in the patients perception since the PROMs do not differ between treatment groups.

## Data Availability

The data that support the findings of this study are not openly available due to reasons of sensitivity and are available from the corresponding author upon reasonable request.
